# Therapeutic strategies for critically ill patients with COVID-19

**DOI:** 10.1186/s13613-020-00661-z

**Published:** 2020-04-20

**Authors:** Lei Li, Ranran Li, Zhixiong Wu, Xianghong Yang, Mingyan Zhao, Jiao Liu, Dechang Chen

**Affiliations:** 1 Department of Critical Care Medicine, Ruijin Hospital, Shanghai Jiao Tong University School of Medicine, Shanghai, 200025 People’s Republic of China; 2 Department of Surgical Intensive Care Unit, Huadong Hospital Affiliated to Fudan University, Shanghai, 200040 People’s Republic of China; 3 Department of Critical Care Medicine, Zhejiang Provincial People’s Hospital, Hangzhou, Zhejiang 310014 People’s Republic of China; 4 Department of Critical Care Medicine, The First Hospital Affiliated to Harbin Medical University, Harbin, 150001 People’s Republic of China; 5 Department of Critical Care Medicine, Ruijin North Hospital, Shanghai Jiao Tong University School of Medicine, Shanghai, 201800 People’s Republic of China

**Keywords:** Coronavirus, 2019-nCoV, Antiviral therapy, Adjunctive intervention, Respiratory support

## Abstract

Since the 2019 novel coronavirus disease (COVID-19) outbreak originated from Wuhan, Hubei Province, China, at the end of 2019, it has become a clinical threat to the general population worldwide. Among people infected with the novel coronavirus (2019-nCoV), the intensive management of the critically ill patients in intensive care unit (ICU) needs substantial medical resource. In the present article, we have summarized the promising drugs, adjunctive agents, respiratory supportive strategies, as well as circulation management, multiple organ function monitoring and appropriate nutritional strategies for the treatment of COVID-19 in the ICU based on the previous experience of treating other viral infections and influenza. These treatments are referable before the vaccine and specific drugs are available for COVID-19.

## Introduction

In late December 2019, a group of patients with pneumonia of unknown cause were confirmed to be infected with a novel coronavirus (2019-nCoV) in Wuhan, China. The 2019-nCoV has now infected tens of thousands of people in China and has spread rapidly around the globe [1]. The World Health Organization (WHO) has declared coronavirus disease 2019 (COVID-19) as a Public Health Emergency of International Concern and released interim guidelines on patient management [2]. Due to the severity and the spreading of COVID-19 (novel coronavirus pneumonia, NCP), the Chinese government and the medical institutions have executed strict strategies to control the influence of this epidemic [3]. Until the end of February, the epidemic has been controlled to a great extent nationally. In Wuhan, the situation tends to be stable while a high proportion of critically ill patients are still under treatment of intensive care.

## The identification of 2019-nCoV

Coronaviruses (CoVs) are enveloped viruses with a single positive-stranded RNA genome (~ 26–32 kb in length). CoVs mainly cause respiratory tract infections and some strains have high infectivity and mortality as well as heavy damage on public health, such as severe acute respiratory syndrome (SARS) and Middle East respiratory syndrome (MERS). The 2019-nCoV is a β-CoV of group 2B with over 70% similarity in genetic sequence with SARS-nCoV [4, 5]. The latest version of diagnosis and treatment plan pointed out that the main transmission route is droplet transmission and close contact transmission. In addition, there are risks of airborne spread of 2019-nCoV during aerosol-generating medical procedures in specific circumstances [6, 7].

## Diagnosis and clinical classification of COVID-19

The clinical manifestations of COVID-19 are fever, headache, dry cough, with radiological evidence of viral pneumonia. In severe cases, dyspnea usually occurs about 1 week after the disease onset and some patients can rapidly progress to acute respiratory distress syndrome (ARDS), septic shock, refractory metabolic acidosis, and coagulation disorders [8]. In addition, the clinical features of asymptomatic cases are not obvious except for the positive nucleic acid in oropharyngeal swabs. Asymptomatic cases also have the risk, although weak, of transmission. Respiratory viral infection can cause severe illness, especially in the elderly and persons with co-morbidities [9].

According to the latest version of diagnosis and treatment guidelines, confirmed cases infected with 2019-nCoV are classified to have severe illness once complying with one of the following symptoms: (1) anhelation, respiratory rate ≥ 30 times/min; (2) oxygen saturation at rest ≤ 93%; (3) PaO2/FiO2 ≤ 300 mmHg; and classified to be the critical/life-threatening illness once complying with one of the following symptoms: (1) respiratory failure, mechanical ventilation needed; (2) shock; (3) other organ dysfunction syndrome and requirement of intensive care unit admission.

The progress of the severe illness with COVID-19 is usually rapid and there is no clear separation between the severe illness and the critical illness. Therefore, patients of these two classes are combined to be the critical illness, which is helpful for health care workers to diagnose and treat patients with intensive care and resources at the early stage of the critical illness.

The diagnostic evidences for ICU admission according to the previous experience in the treatment of SARS include old age (> 60 years old), presence of co-morbidities (particularly, diabetes mellitus, hepatic or cardiac disease), and elevated lactate dehydrogenase levels on admission to hospitals [10, 11].

## Therapeutic strategies for COVID-19 in the ICU

2019-nCoV invades through the respiratory mucosa and infects other cells, inducing cytokine storm systemically [12]. Some patients may progress rapidly with ARDS, disseminated intravascular coagulation (DIC), septic shock, and eventually multiple organ failure [13]. Therefore, early identification and timely treatment of critical cases is of crucial importance. Evidence-based therapy and supportive care in ICU is the mainstay for the management of severe and life-threatening illness of COVID-19.

The severe and critical illnesses with COVID-19 should be treated in ICU in the hospital with nosocomial infection control. Strict volume management, multi-organ function evaluation, critical care of the nutritional assessment/appropriate nutritional support are essential for these patients in ICU. In addition, attention should be paid to bedbound patients to prevent deep vein thrombosis.

## Antiviral therapy

At present, there is no antiviral treatment with confirmed effectiveness for COVID-19. Available drug options that come from the clinical experience of treating SARS, MERS and other previous influenza virus have been used for the treatment of COVID-19 patients. Although these antiviral drugs are promising in the treatment of COVID-19, it should be kept In mind that: (1) the adverse effects of the drugs need to be monitored in clinic, (2) the effects of these drugs on critically ill patients still need to be clarified, (3) the potential mutation of the coronavirus may lead to the drug resistance of the virus.

### Fabiravir and ribavirin

Nucleoside analogs have a broad-spectrum antiviral effect via the mechanisms of lethal mutagenesis, chain termination, and inhibition of nucleotide biosynthesis. Fabiravir and ribavirin are representative drugs of nucleoside analogs and exhibit the antiviral effect by inhibiting nucleotide biosynthesis. It has been demonstrated that the combination of fabiravir and oseltamivir in the treatment of severe influenza may accelerate clinical recovery than oseltamivir alone [14]. In addition, it has been reported that the combination of ribavirin and interferon alpha (IFN-⍺) significantly reduced the 14-day mortality of critically ill patients infected with MERS, although the 28-day mortality was not affected [15]. Ribavirin and IFN-⍺ were also used in the treatment for SARS. However, ribavirin might have side effects such as anemia and liver injury, and IFN-⍺ may not improve the patients’ outcome [16]. Therefore, the use of ribavirin and IFN-⍺ in the treatment of COVID-19 needs to be further elucidated by clinical studies.

### Lopinavir/ritonavir

Lopinavir/ritonavir is a protease inhibitor in the treatment of HIV infection. Lopinavir/ritonavir showed the antiviral activity by inhibiting the replication of coronavirus in vitro. It has been reported that the combination of lopinavir/ritonavir with ribavirin could lower the risk of ARDS compared with ribavirin alone [17]. Most recently, the randomized clinical trial of lopinavir/ritonavir (400 mg/100 mg, twice-daily for 14 days) in the treatment of COVID-19 by Cao et al. has shown that in hospitalized adult patients with severe COVID-19, no beneficial effect was observed with lopinavir/ritonavir treatment compared with standard care group [18]. The adverse effects of lopinavir/ritonavir treatment include anorexia, nausea, abdominal discomfort, diarrhea, or acute gastritis. Moreover, the risk of hepatic injury, pancreatitis, more severe cutaneous eruptions, as well as the drug interactions due to CYP3A inhibition has been observed in the clinical trial, which arouses concern about the use of higher or prolonged dose regimens for outcome improvement [18]. In addition, serious complications such as acute kidney injury and secondary infection were fewer than in those not receiving treatment. Future trials with severe illness might help to elucidate the possibility of benefit of lopinavir/ritonavir treatment.

### Remdesivir

Remdesivir (GS-5734) is a new nucleoside analog and has been recognized as a potential and promising antiviral drug against a wide array of RNA viruses, including SARS/MERS-CoV. It is currently under clinical development for the treatment of Ebola virus infection [19]. Remdesivir potentially inhibits the RNA-dependent RNA polymerase from MERS-CoV, reduces virus replication, decreases the virus titer in mouse lungs infected with MERS-CoV, and improves the lung tissue damage [20, 21]. The antiviral activity of remdesivir and IFN-beta was found to be superior to that of the combination of lopinavir/ritonavir and IFN-beta against MERS-CoV [22]. A randomized, controlled trial has reported that the prolonged use of remdesivir in the treatment of Ebola virus disease (EVD) is safe [19], and no adverse events have been observed [23]. As a candidate drug that has not been approved, information about the side effects of remdesivir has not been reported yet. At present, two randomized, controlled, double-blind clinical trials are ongoing to evaluate the efficacy and safety of remdesivir (200 mg loading dose on Day 1, followed by 100 mg i.v. once-daily maintenance dose for 9 days) in hospitalized patients with mild/moderate or severe COVID-19 respiratory disease [24, 25]. The results of these clinical trials may open the window for effective antiviral therapy for such an epidemic infectious disease.

### Arbidol

Arbidol is a small indole-derivative molecule and is approved for the prophylaxis and treatment of influenza and other respiratory viral infections [26]. It also showed inhibitory activity against other viruses, enveloped or not, responsible for emerging or globally prevalent infectious diseases such as hepatitis B and C [27]. In addition, arbidol has been reported to have antiviral activity against the pathogen of SARS, and the effect of arbidol mesylate—a derivative of arbidol, was almost five times higher than arbidol in reducing the reproduction of SARS in cells in vitro [28]. It has been claimed that arbidol was effective against 2019-nCoV in vitro [29]. A randomized multicenter controlled clinical trial of arbidol in patients with 2019-nCoV is in progress in China [30].

### Chloroquine and hydroxychloroquine

It is known that angiotensin-converting enzyme-2 (ACE2) as a membrane protein is a functional receptor of SARS-CoV and it can facilitate virus entry into the cells by binding to the spike (S) protein of the virus, which mediates the fusion of viral and host membranes [31–33]. Therefore, it may be of importance to block the binding of S protein to ACE2 to treat viral infection, such as SARS-CoV [34]. Chloroquine is a 9-aminoquinoline known since 1934, the sulfate and phosphate salts of which have both been commercialized as widely used antimalarial and autoimmune disease drugs. Chloroquine also shows broad-spectrum antiviral effects [35]. It was found to be a potent inhibitor of SARS-CoV infection due to its inhibitory effect on ACE2 [36]. It has been demonstrated that 2019-nCoV enter the epithelial cells of oral mucosa via the essential receptor ACE2 [37], and chloroquine can function at both entry and post-entry stages of 2019-nCoV infection [38]. Besides the antiviral activity, chloroquine has an immune-modulating activity, which may synergistically enhance its antiviral effect in vivo. Recently, Wang et al. have demonstrated that chloroquine is highly effective in the control of 2019-nCoV infection in vitro and is suggested to be assessed in human patients suffering from COVID-19 [38]. In addition, the results from more than 100 COVID-19 patients have indicated that chloroquine phosphate is superior to the control treatment in inhibiting the exacerbation of pneumonia, improving lung imaging, promoting virus negative conversion, and shortening the disease course [39]. However, attention should be paid to the potential detrimental effects of chloroquine observed in previous attempts to treat viral diseases. At present, the clinical trials to evaluate the efficacy and safety of chloroquine in the treatment of COVID-19 is ongoing [40]. The use of chloroquine in the treatment of COVID-19 should refer to the most recent announcements if any.

In addition, hydroxychloroquine is a 4-aminoquinoline derivative antimalarial drug. Hydroxychloroquine is an immunosuppressive drug with mature clinical application in the treatment of rheumatic immune diseases such as rheumatoid arthritis and systemic lupus erythematosus [41, 42]. It has been found to be more potent than chloroquine in inhibiting 2019-nCoV in vitro. Hydroxychloroquine sulfate 400 mg given twice daily for 1 day, followed by 200 mg twice daily for another 4 days is recommended in the treatment COVID-19 [43]. At present, the clinical evaluation of hydroxychloroquine in the treatment of COVID-19 is in progress [44], which might shortly provide preliminary results about the effectiveness of hydroxychloroquine.

## Antibacterial therapy

Patients with pneumonia, especially in severe condition, may encounter with co-infection or cross-infection of bacterial pathogens, for instance *staphylococcus aureus*, during medical treatment in the hospital. Considering the high incidence of bacterial infection for critically ill patients with COVID-19, it is essential to test the kinetics of procalcitonin (PCT) and C-reaction protein (CRP) in COVID-19 patients for timely diagnosis and intervention of bacterial infection. According to the recent 2019 ATS/IDSA clinical practice guidelines, besides antiviral treatment for patients with viral-infected pneumonia, clinicians should empirically give antibacterial therapy to patients that initially have severe diseases (extensive pneumonia, respiratory failure, hypotension, and fever), or deteriorate after initial improvement, or fail to improve after 3 to 5 days of antiviral treatment [45]. Thus, antibiotic treatment is recommended in the treatment of COVID-19 patients based on the evidence of bacterial infection. The blind and inappropriate use of antibiotics, especially the broad-spectrum antibiotics, should be avoided.

## Adjunctive interventions

Immune disorders have been observed in the treatment of patients with COVID-19. The virus infection activates the immune cells, leading to cytokine storm which is associated with disease severity [46]. On the other hand, the critical illness with COVID-19 mainly affect elders or people with chronic diseases, some of whom have very low number of lymphocytes, especially CD4+ T cells, implying deficiency of immune system. Therefore, to modulate the immune responses, a variety of pharmacologic agents have been proposed [47, 48].

### Corticosteroids

The use of corticosteroids in the treatment of ARDS is controversial. Observational data in SARS suggest that immunomodulation with regimens of high-dose methylprednisolone might be helpful in modulating inflammatory responses and lung damage [49, 50]. On the other hand, other studies showed that use of steroids is associated with increased risk for bacterial infection, increased mortality, and even antiviral resistance in influenza-associated pneumonia or ARDS [51–54]. Moreover, multiple studies have reported that corticosteroid treatment is associated with delayed viral shedding in hospitalized patients without significant change in 90-day mortality [55–57]. Corticosteroid therapy in patients with MERS was shown to be not associated with a difference in mortality, but associated with delayed MERS coronavirus RNA clearance [57].The early use of parenteral glucocorticoids therapy for fever reduction and pneumonia prevention has been shown to increase the risk for critical disease or death from H1N1 infection [58].

The currently limited clinical research does not support the use of corticosteroids in the treatment of ARDS in COVID-19 patients to improve the outcome of patients. The WHO recommended that corticosteroids should not be used in the treatment of viral pneumonia or ARDS. There is no convincing proof for the therapeutic benefits of corticosteroids in the treatment of COVID-19, which still need to be demonstrated in clinical research.

### Thymosin alpha-1

Thymosin alpha-1 is a thymic peptide hormone with significant benefits in restoring the homeostasis of the host immune system [59]. It is chemically synthesized and used in diseases with impaired immune system [60]. It has been reported that the low lymphocyte count is associated with the poor prognosis of septic patients. The use of thymosin alpha-1 therapy in combination with conventional medical therapies was effective in improving clinical outcomes and reducing mortality in severe sepsis [61]. In addition, thymosin alpha-1 can enhance the immune responses of SARS patients and help to limit the spreading of SARS [62]. Therefore, although there is no clinical evidence showing the beneficial effects of thymosin alpha-1 in COVID-2019, it has been recommended to be used for some patients to enhance cellular immunity for the resistance of viral infection.

### Cyclosporine A

Cyclosporine A is widely used in transplantation and autoimmune disorders due to its immunosuppressive effect. Cyclophilin A as a key member of immunophilins is the cellular receptor for cyclosporine A [63]. The inhibition of cyclophilins by cyclosporine A could block the replication of coronavirus, including SARS-CoV [64]. Therefore, non-immunosuppressive derivatives of cyclosporine A might serve as broad-range CoV inhibitors applicable against emerging virus like 2019-nCoV, which still needs to be confirmed by clinical studies in the future.

### Interferons

There are two types of interferons (IFNs), type I IFNs and type II IFNs. It has been demonstrated that type I IFNs can inhibit the replication of both SARS and MERS-CoV [65, 66]. Kuri et al. have reported that IFN transcription was blocked in tissue cells infected with SARS-CoV and cells infected with SARS-CoV were able to partially restore their innate immune responsiveness to SARS-CoV after priming with small amounts of IFNs [67]. Moreover, in patients with severe MERS-CoV infection, the combination of IFN-alpha-2a with ribavirin was shown to improve survival [66]. Recently, the combination of remdesivir and IFN-beta was shown to have significant antiviral activity [22].

### Gammaglobulin

Intravenous gammaglobulin is considered as the safest immunomodulating drug available for the treatment of severe infection and sepsis. It has high titers of neutralizing antibodies against broad-spectrum virus, bacteria, and other pathogens, and can modulate the host immune responses in several ways. However, a large-scale multicenter randomized placebo-controlled trial did not show improved survival with intravenous gammaglobulin in severe sepsis [68]. Moreover, a Cochrane review showed that intravenous gammaglobulin did not reduce the mortality of septic patients [69]. Therefore, there is no convincing argument to recommend intravenous gammaglobulin in the treatment of 2019-nCoV.

### Tocilizumab

One of the most important mechanism underlying the deterioration of COVID-19 is cytokine storm characterized by elevated levels of IL6, IFN- and other cytokines, which will lead to ARDS or even multiple organ failure [70]. Tocilizumab is a recombinant humanized monoclonal antibody binding to IL6 receptor and inhibiting its signal transduction. Tocilizumab has been used in the treatment for rheumatoid arthritis (RA) [71]. Moreover, tocilizumab has been reported to be effective against cytokine release syndrome induced by CAR-T cell infusion against B cell acute lymphoblastic leukemia [72]. In Diagnosis and Treatment Guidelines of NCP (trial version 7.0) [73], tocilizumab is recommended for the immunotherapy of patients with extensive lung lesions and severe cases that show an increased level of IL6 in laboratory testing. The efficacy of tocilizumab in COVID-19 patients still needs to be investigated.

## Chinese traditional medicine

In 2003, Chinese traditional medicine was used to prevent and treat SARS [74]. In 2009, during the pandemic of H1N1 influenza, the Traditional Chinese Medicine of China issued a Chinese traditional medicine prevention program, which included several Chinese herbal medicine formulae for the prevention of infection of adults and children. ShuFengJieDu capsules and Lianhuaqingwen capsules have also played a role in the prevention and treatment of new respiratory infectious diseases such as influenza A (H1N1) [75, 76]. Some studies have confirmed that Yupingfeng powder has antiviral, anti-inflammatory and immunoregulatory effects [77]. A multicenter, large-scale, randomized trial found that Yinqiao powder plus another heat-clearing formula could reduce time for fever resolution in patients with the H1N1 influenza virus infection [78].

It is suggested that high-risk populations exposed to COVID-19 patients, including medical staff, family members, and other people who are in close contact with COVID-19 patients, as well as residents living in COVID-19 outbreak areas, might benefit from taking Chinese traditional medicine formulae for prevention. However, the efficacy and safety of these Chinese traditional medicine formulae in COVID-19 need to be further confirmed by clinical trials.

## Convalescent plasma

The convalescent plasma derived from the patients with antibodies against 2019-nCoV can be effective in reducing the mortality rate of critically ill patients with infectious disease [79]. Convalescent plasma has been found to have an immunotherapeutic potential for the treatment of MERS, SARS and Ebola virus disease [80–82]. The explanation for the efficacy of convalescent plasma therapy is that antibodies from convalescent plasma might suppress viremia via free viral clearance, blockade of new infection, as well as the acceleration of infected cell clearance [83]. In addition, the use of high-titer MERS serum from camel could significantly improve the histology of lung damage and increase the clearance of MERS-CoV in mice [84]. Moreover, the use of convalescent plasma or serum was also suggested by WHO under Blood Regulators network when vaccines and antiviral drug was unavailable for an emerging virus. Evidence shows that convalescent plasma therapy is not associated with the occurrence of severe adverse events [85]. Convalescent plasma, if available, can be used for the treatment of critically ill patients with COVID-19 after the evaluation of the valence of antibody. It is worthwhile to test the efficacy and safety of convalescent plasma transfusion in COVID-19 patients.

## Respiratory supportive strategies

A meta-analysis showed that among patients with 2019-nCoV infection, the incidence of ARDS is approximately 15% [12]. Moreover, between 50% to 85% of patients admitted to ICU have hypoxemia/or development of respiratory exhaustion [86]. Therefore, timely and effective respiratory support can contribute to reduce complications and improve the survival of such critically ill patients.

### Oxygen therapy and mechanical ventilation

Oxygen therapy, high-flow nasal cannula, and non-invasive ventilation may reduce the need of endotracheal intubation and decrease ventilator-associated complications and mortality. However, several studies have reported that the failure of non-invasive ventilation was up to 85% in which invasive ventilation was ultimately required in the treatment of severe influenza A (H1N1) in Canada [87]. Non-invasive ventilation may be effective and safe for some patients, whereas it might increase virus transmission to health care workers because of risk for infected aerosol generation. Therefore, for the treatment of 2019-nCoV, non-invasive ventilation may be used in selected patients in early stages with milder acute hypoxemic respiratory failure [88]. While for critically ill patients, the effectiveness of transitionally oxygen therapy, such as respiratory status and oxygen index (PO2/FiO2), needs to be closely monitored and it should be switched to mechanical ventilation when necessary.

High-flow nasal cannula has emerged as an alternative to non-invasive ventilation to prevent intubation and reduce mortality in patients with acute hypoxemic respiratory failure [89]. High-flow nasal cannula has been reported to significantly reduce 90-day mortality of community-acquired pneumonia compared with standard oxygen or non-invasive ventilation [89]. Hui et al. have shown that the breath dispersion distance is limited therefore lowering the risk of air transmission. However, the loose connection of the cannula with nasal obstruction can significantly increase the dispersion distance [90]. Wearing masks (particularly N95) can effectively reduce the breath dispersion distance during high-flow nasal ventilation to prevent nosocomial transmission [91, 92]. In addition, high-flow nasal cannula might increase virus transmission risk due to aerosol generation. Therefore, staff protection of health care workers is critical.

Mechanical ventilation for patients with severe ARDS should be managed with lung-protective strategies to minimize ventilator-associated lung injury and to improve survival. The approach to minimize ventilator-associated lung injury and to improve survival includes: ventilation with low tidal volumes (4 to 8 mL/kg of predicted body weight), targeting plateau pressure (< 30 cmH_2_O) [93], and minimizing the inspired oxygen concentration to decrease oxygen toxicity. High positive end-expiratory pressure (PEEP) can reduce the need for high FiO2 by improving gas exchange and lung compliance, whereas too high PEEP may lead to lung overdistension and hemodynamic instability. Optimal PEEP which can be titrated by pressure–volume curve, oxygenation, stress index, electrical impedance tomography (EIT), ultrasound, and some other clinical parameters is associated with improved survival rate among severe ARDS patients [9].

Lung recruitment needs to be evaluated for mechanically ventilated patients when uncorrectable hypoxemia occurs, and lung recruitment should be executed for patients whose lungs can restore aeration. CT, EIT, ultrasound, and other bedside techniques should be used to evaluate lung recruitability before lung recruitment.

For critically ill patients managed with mechanical ventilation, excessive spontaneous breathing due to stretching may lead to lung injury. Therefore, neuromuscular relaxant may be used to control the spontaneous breathing and protect the lung.

### Prone positioning ventilation

Prone positioning ventilation is a technique that often improves oxygenation in ARDS, possibly through improvements in ventilation–perfusion matching, the uniformity of ventilation, and gravity-related atelectasis. Prone ventilation was used in mechanically ventilated SARS patients without enough data to draw any conclusion with regard to its efficacy [86]. Although prone ventilation showed no improvement in survival or organ dysfunction overall, it might be beneficial for patients with severe ARDS. A multicenter RCT demonstrated that early application of prone positioning in patients with severe ARDS resulted in decreased mortality [94]. In addition, the use of prone positioning in patients with H7N9 influenza-induced severe ARDS was shown to be related with improved oxygenation, sustained after returning to a supine position, and with decreased carbon dioxide retention [95]. Generally speaking, prone positioning ventilation for no less than 12 h daily is a relatively safe procedure that rarely worsens a patient’s respiratory status. It can be thus recommended for the treatment of 2019-nCoV-induced severe ARDS.

### Extracorporeal membrane oxygenation (ECMO)

ECMO has become the important life-support strategy for the standardized treatment of ARDS patients. ECMO should be considered as early as possible when the lung recruitment and prone positioning ventilation show to be ineffective.

Observational studies have reported that patients with ARDS induced by H1N1 influenza showed lower hospital mortality with transfer to an ECMO center compared with matched non-ECMO-supported patients [96]. The use of ECMO also showed survival benefit in patients with severe MERS [97]. ECMO tends to improve patient outcomes when used among those with limited organ failures and good pre-morbid functional status [9]. Substantial proportion of critically ill patients with COVID-19 appear to have developed cardiac arrhythmias or shock [13], and may need ECMO support. However, for those who will develop septic shock or refractory multiple organ failure, ECMO is not suggested due to its less benefit.

ECMO is a resource-intensive, highly specialized and expensive form of life support with potential for significant complications such as hemorrhage and nosocomial infection. Therefore, the use of ECMO should be strictly limited in the treatment of COVID-19. Moreover, since the number of critically ill patients is still increasing and the resource of ECMO is finite, judgment is needed to decide when ECMO may be worthwhile and when it may not. Support with ECMO is supposed to be for the most critically ill patients in regions with extensive resources for this therapy [98].

## Circulatory support and fluid management

For patients with ARDS, restrictive and timely fluid resuscitation is associated with better oxygenation and lower mortality. Aggressive fluid administration may worsen oxygenation and ventricular dysfunction, which may result in longer duration of mechanical ventilation and even mortality. Therefore, it is necessary to assess fluid responsiveness and to evaluate ventricular function during fluid resuscitation. Conservative fluid administration while maintaining adequate mean arterial pressure and organ perfusion with the appropriate use of diuretics and vasopressors is of importance [99].

## Multiple organ functional support and nutrition strategy

According to the latest epidemiological report, the incidence for the critically ill patients to develop multiple organ dysfunction syndrome is up to 11% [48]. COVID-19 may be combined with other organ injuries, including liver injury, cardiac dysfunction, coagulopathy, which may need the routine functional support for critically ill patients in ICU.

Moreover, all the critically ill patients with COVID-19 admitted into ICU have negative nitrogen balance and malnutrition [12, 46], which has been considered as a contributing factor to the emergence of viral infectious diseases. Therefore, appropriate nutritional strategy is pivotal for the treatment of critical illnesses when necessary.

## Summary

There are no specific antiviral drugs or vaccines for 2019-nCoV at present. Therefore, it is important to enhance the host immune response against the infection with 2019-nCoV. All of the drug options are based on the experience treating SARS, MERS or some other previous influenza viruses. The efficacy of existing drugs as well as adjunctive pharmacologic interventions in the treatment of critical ill patients with COVID-19 warrants further verification in clinical research. To completely stop the epidemic spreading of COVID-19, a vaccine for 2019-nCoV is urgently needed. Besides enhancing the host immune responses against viral infection, appropriately respiratory supportive strategies, monitoring and support of multiple organ function, modulating the immune status and inflammatory responses individually, as well as the prophylaxis and treatment of complications are all important guarantee for the recovery of critically ill patients with COVID-19. For a better understanding of this novel virus, more research needs to be done to get optimal strategies for the treatment of COVID-19.

## Data Availability

Not applicable.
